# Semantic Knowledge Representation for Strategic Interactions in Dynamic Situations

**DOI:** 10.3389/fnbot.2020.00004

**Published:** 2020-02-13

**Authors:** Carlos Calvo Tapia, José Antonio Villacorta-Atienza, Sergio Díez-Hermano, Maxim Khoruzhko, Sergey Lobov, Ivan Potapov, Abel Sánchez-Jiménez, Valeri A. Makarov

**Affiliations:** ^1^Facultad de CC. Matemáticas, Instituto de Matemática Interdisciplinar, Universidad Complutense de Madrid, Madrid, Spain; ^2^Biomathematics Unit, Faculty of Biology, Complutense University of Madrid, Madrid, Spain; ^3^N. I. Lobachevsky State University, Nizhny Novgorod, Russia

**Keywords:** cognitive maps, manipulation of objects, dynamical systems, semantic description, neural networks

## Abstract

Evolved living beings can anticipate the consequences of their actions in complex multilevel dynamic situations. This ability relies on abstracting the meaning of an action. The underlying brain mechanisms of such semantic processing of information are poorly understood. Here we show how our novel concept, known as time compaction, provides a natural way of representing semantic knowledge of actions in time-changing situations. As a testbed, we model a fencing scenario with a subject deciding between attack and defense strategies. The semantic content of each action in terms of lethality, versatility, and imminence is then structured as a spatial (static) map representing a particular fencing (dynamic) situation. The model allows deploying a variety of cognitive strategies in a fast and reliable way. We validate the approach in virtual reality and by using a real humanoid robot.

## 1. Introduction

Efficient object manipulation is simultaneously one of the most apparent features of humans' daily life and one of the most challenging skills that modern humanoid robots largely lack (see e.g., Calvo et al., 2018b; Billard and Kragicet, 2019 and references therein). The sensory-motor abilities ordinarily exhibited by humans may appear dull at first glance. However, children spent years to acquire adult-equivalent skills in manipulation (Thibaut and Toussaint, 2010). Therefore, such simple-but-difficult tasks possess vast intrinsic complexity, which impedes robots to mimic even basic human abilities in real-life scenarios.

Modern robots are capable of manipulating objects in repetitive and controlled conditions, e.g., in industrial assembly setups. In such tailor-made scenarios, a purely programmatic approach to the problem of limb movement works rather well (Choset et al., 2005; Patel and Shadpey, 2005). The development of adaptive techniques, the use of control theory, and learning in neural networks made it possible to adjust the robot's trajectories to comply with some degree of uncertainty. Nowadays, robots can retrieve objects at different locations, e.g., from a conveyer belt or even catch fast-moving objects (Kim et al., 2014; Nguyen et al., 2016; Bouyarmane et al., 2018; Mason, 2018). There is a growing body of approaches addressing the problems of a robust prediction of trajectories of objects, fast calculation of feasible postures and movements of limbs through, e.g., splines, etc. (Riley and Atkeson, 2002; Aleotti and Caselli, 2006; Xiao et al., 2016).

Although robots strive to dexterous object handling and gradually improve skills in orientation in space (Billard and Kragicet, 2019), they still undergo difficulties in handy and safe interactions with humans in time-evolving situations. Such cooperation requires the implementation of motor cognition at different levels of decision-making, including the abstract one (Villacorta-Atienza and Makarov, 2013). The latter, in particular, can be approached through studies of brain structures and functions involved in cognitive phenomena (Sporns, 2011; Calvo et al., 2020).

The remarkable human capacity to actuate in complex situations relies in part on semantic memory (for a review, see Binder and Desai, 2011). Models of semantic memory have seen an impressive improvement that has dramatically advanced our understanding of how humans create, represent, and use meanings from experiences (for a review, see Jones et al., 2015). The semantic organization of concepts and features is much more economical in terms of the memory capacity and ability of generalization. This advantage enables an efficient building of unexpected compound strategies and new knowledge.

Semantic memory uses the features and attributes of experiences that define concepts, and allow us to efficiently retrieve, act upon, and produce information in the service of thought and language. While the application of this methodology to simple concepts made of items (e.g., a lion, a tree, a table) and their features (e.g., wild, green, long) was hugely successful (Ralph et al., 2017), it has been used in studies of motor skills to a much lesser extent.

We will call a motor-motif a particular movement of a limb or a body in a specific time window. Then, a sequence of motor-motifs composes a behavior that can be arbitrarily complex (Calvo et al., 2018a). Each motor-motif is a function of space and time. The internal representation of such spatiotemporal objects in the brain is a challenging open problem (Livesey et al., 2007; Kraus et al., 2013; Bladon et al., 2019). How the brain generates concepts, and thus semantic memories from movement experiences, is largely unknown. To solve this puzzle, in our previous works, we proposed a theoretical hypothesis called time compaction (Villacorta-Atienza et al., 2010, 2015), which recently received experimental support (Villacorta-Atienza et al., 2019).

Time compaction states that when dealing with time-changing situations, the brain does not encode time explicitly but embeds it into space. Then, a dynamic situation (i.e., a spatio-temporal structure) is transformed into a purely static object, the so-called generalized cognitive map (GCM). A GCM, in particular, contains images of motor-motifs in the form of points in some configuration space. Such an enormous dimension reduction (compaction of time) significantly reduces brain resources required for the planning of trajectories in complex situations, including motor interactions of humans (Villacorta-Atienza et al., 2015). It also enables building concepts out of motor-motifs by using the principle of the high-dimensional brain (Calvo et al., 2019; Gorban et al., 2019, 2020; Tyukin et al., 2019).

Standard cognitive maps (CMs) are abstract internal representations of static situations in the brain (Tatler and Land, 2011; Schmidt and Redish, 2013; Noguchi et al., 2017). Such representations enable navigation in static environments, and, to some extent, can be compared to a modern GPS providing the ability to plan routes using a map with roads and obstacles (e.g., buildings) to be avoided (Schmidt and Redish, 2013). Similar to a standard CM, a GCM is also an abstract description of the environment, but it extends CMs into dynamic situations. Thus, the GCM approach allows selecting different strategies or motor-motifs to navigate in dynamic situations by using special maps.

Hypothetically, the GCM approach enables building semantic motor memory out of motor-motifs embedded as points into GCMs. However, no successful attempts have been made yet. In this work, we develop a novel approach to constructing behaviors based on the semantic description of motor-motifs emerging from GCMs. The method is illustrated in the simulation of the combat sport of fencing and further validated experimentally on a humanoid robot.

## 2. Materials and Methods

We begin with a practical example of the combat sport of fencing that will help us introduce the main idea of building cognitive strategies. Fencing is a highly demanding sport based on the perfect coordination of fast movements, where points are scored by hitting an opponent by the tip of a foil. Each dynamic, i.e., a time-evolving situation, gives rise to an internal brain model. Such a model includes relevant spatial and temporal aspects of the situation (Kraus et al., 2013). Our goal is to provide a semantic description of the strategic planning based on GCMs and motor-motifs. In the following subsections, we thus briefly summarize the GCM concept, introduce the configuration space of a manipulator, discuss how a GCM and motor-motifs can be constructed in the configuration space, and provide the robot design for validating the theory.

### 2.1. Generalized Cognitive Maps

The concept of GCMs stems from standard cognitive maps. In time-changing situations, e.g., while navigating in a crowd, standard CMs are not suitable since the maps continuously change in time. The neural and functional mechanisms behind human decision making in time-changing situations are mostly unknown. Recently, a hypothesis that the brain entangles spatial and temporal dimensions in a single entity has been proposed (Villacorta-Atienza et al., 2010; Buzsaki and Llinas, 2017), which in the end gave rise to the GCM concept.

According to the hypothesis, the brain transforms “time into space” (Villacorta-Atienza et al., 2010; Villacorta-Atienza and Makarov, 2013). Such a functional mechanism, called time compaction, allows representing a dynamic situation as a static map, similar to a standard CM. The resulting generalized cognitive map also has steady obstacles and can be used to trace routs for navigation. A standard CM and a GCM of a static scene are equivalent, i.e., the static obstacles appear in both maps at the same places.

The advantage of GCMs is the projection of objects moving in the environment into a map as virtual obstacles. Such projection or time compaction occurs by identifying places of the potential collisions of the subject with moving objects. It is achieved by predicting and matching all trajectories of the moving elements and the subject. The latter is accomplished by a wave process simulating all possible movements simultaneously (Villacorta-Atienza et al., 2010). The places of potential collisions become virtual obstacles that subject should stay away to avoid crashes while navigating.

Time compaction is useful for navigation in different dynamic situations (Villacorta-Atienza et al., 2015). However, its power goes far beyond effective or “applied” cognition. The static representation of the subject's actions as mere points in a configuration space enables building memories of static images of motor-motifs, instead of memorizing the whole spatiotemporal situations (Villacorta-Atienza and Makarov, 2013). Then, the subject can establish causal relationships among such images and build high-level cognitive strategies in complex dynamic situations. Below we develop a model for semantic knowledge representation by means of linking images of motor-motifs as pieces in the Tetris-like game on the abstract cognitive level.

### 2.2. Model of Fencing in Hand-Space

**Figure 1:**
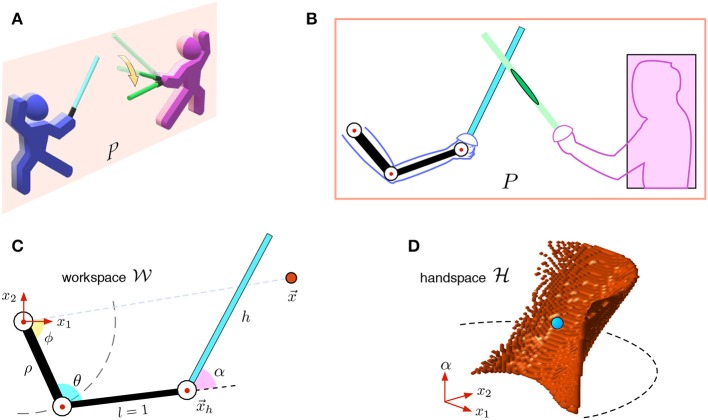
Model of fencing: from workspace to hand-space. **(A)** Sketch of combat of two fencers. The cognitive fencer (the subject colored in blue) is aligned within plane *P*. The opponent (in pink) moves the foil along the yellow arrow crossing the plane. **(B)** In plane *P*, the subject's upper limb has three joints (3 DoF), while the opponent is reduced to a rectangle. **(C)** Kinematic model of the subject's limb and foil in the workspace W. Red dot at $\vec{x}$ is a object point. **(D)** The model in the hand-space H. The subject's limb and the foil are reduced to a single point (in blue), while the object point is extended to a surface (in red).

Figure 1A sketches a typical situation of combat of two fencers. During training, one of the fencers (a learner, Figure 1A in blue) responds to the pre-designed movements of the other (a teacher, in pink). In what follows, we will model the actions of the learning subject.

#### 2.2.1. Kinematic Model in Workspace

We consider the fencer colored in blue in Figure 1A as a cognitive subject. Let *P* be the main plane aligned with the subject's upper limb and the foil. For simplicity, we assume that foils' tips can contact with the fencers' bodies within this plane only. The subject's limb and the foil can be described as a three-segment mechanical system with tree joints rotating within certain physiological limits (Figure 1B). The opponent's body in plane *P* can be represented by the minimal rectangle (colored in pink).

Figure 1C shows the kinematic model of the subject. Without loss of generality, we assume that the length of the subject's forearm is *l* = 1 a.u. The subject's shoulder is fixed at the origin of the (*x*_1_, *x*_2_)-plane, and it is joined to an articulated elbow by a rigid segment of length ρ. The forearm joins the elbow with the hand at ${\vec{x}}_{h}$. The wrist can flex, thus changing the angle of the last segment of length *h*, representing the foil. Besides, we consider a single-point object $\vec{x}$, which can have different semantic meanings, either a target or an obstacle. In numerical simulations and experimental validation, we use ρ = 1 a.u. and *h* = 3 a.u., which is close to the real anthropometry, the robot sizes, and the foil length used in fencing.

All movements of the subject's limb and foil are restricted to a disk of radius (ρ+1+*h*) centered at the origin. We then introduce the workspace (reachable space):

(1) $$W={\bar{ℬ}}_{\rho +1+h}\setminus \{\vec{0}\}\subset {\mathbb{R}}^{2},$$

where ${\bar{B}}_{r}$ denotes a closed disk of radius *r* centered at the origin. The shoulder, elbow, and wrist joints can rotate within specific limits posed onto the angles ϕ, θ, and α (Figure 1C). Thus, we have defined a redundant three degree of freedom (DoF) mechanical system working in a 2D workspace W. For convenience, we also denote by L⊂W the union of the three segments corresponding to the subject's upper arm, forearm, and foil.

#### 2.2.2. Hand-Space Representation

The original procedure of building GCMs (Villacorta-Atienza et al., 2010) assumes that the subject has a rigid body and can be shrunken into a point. The spatial extension and the changing geometry of the subject's limb and foil bring an additional degree of complexity. To resolve this problem, recently, we have proposed a transformation from the workspace to a configuration space that allows extending the GCM-theory into manipulators (Spong et al., 2006; Calvo et al., 2017, 2018b; Villacorta-Atienza et al., 2017). The transformation eliminates the spatial dimensions and rotational degrees of freedom. Then, the equivalent collision space, called the *hand-space*, is given by:

(2) $$H=\left({\bar{ℬ}}_{1+\rho }\setminus {ℬ}_{\text{max}\{0,1-\rho \}}\right)\times J\subset {\mathbb{R}}^{3},$$

where *J* = [α_min_, α_max_] is the feasible interval of the wrist angle α. Without loss of generality, we assume ρ ≥ 1. Then H is a cylinder (or torus) without the central line.

#### 2.2.3. Mapping From Workspace to Hand-Space

The technique of mapping from W to H has been described elsewhere (Calvo et al., 2017, 2018b; Villacorta-Atienza et al., 2017). Here we briefly summarize our earlier results.

##### 2.2.3.1. Shrinkage of limb and foil

The shrinkage of the arm with the foil, i.e., the set L, is given by the following mapping

(3) $$C(L)=({\vec{x}}_{h},\alpha ),$$

which reduces the three-segment mechanical system L in the workspace (black and cyan segments in Figure 1C) to the single point $\left({\vec{x}}_{h},\alpha \right)$ in the hand-space (blue dot in Figure 1D), corresponding to the hand position and the wrist angle in W.

##### 2.2.3.2. Extension of objects

The price to pay by applying the shrinkage (3) is the augmentation of other objects in the hand-space. Let us first consider a single-point object $\vec{x}\in W$ (red dot in Figure 1C). This point is extended to a set of surfaces $E\left(\vec{x}\right)\subset H$ (red area in Figure 1D) corresponding to coincidences of the point object with the three segments of L in the workspace. Thus,

(4) $$E(\vec{x})=E_{1}(\vec{x})\cup E_{2}(\vec{x})\cup E_{3}(\vec{x}),$$

where *E*_1,2,3_ represent the extensions due to collisions of the object with the upper arm, forearm, and foil, respectively. Note that depending on $\vec{x}$, some of *E*_*j*_ can be empty. For example, if $\vec{x}$ is located outside the region accessible by the upper arm (i.e., $\vec{x}\notin {\bar{B}}_{\rho }$, as in Figure 1C), then $E_{1}\left(\vec{x}\right)\;=\;\emptyset$.

When dealing with objects of arbitrary shape, the extension *E* is applied to each $\vec{x}$ over the object's boundary. This generates extended objects wrapping volumes in H. If the object moves in W, then its extension in H changes with time.

**Extension due to collision with upper arm**, ***E***_**1**_. If a point object at $\vec{x}={\left(x_{1},x_{2}\right)}^{T}$ is reachable by the upper arm, then the upper arm segment contacts the object whenever ϕ = 0. Therefore, we get:

(5) $$E_{1}(\vec{x})=\left\{F_{1}(\theta ,\vec{x}):\theta \in [0,\pi ]\right\}\times J,$$

where

(6) $$F_{1}\left(\theta ,\vec{x}\right)=M\left(\begin{array}{c} \rho -\text{cos }\theta \\ \text{sin }\theta \end{array}\right),\;M=\frac{1}{\left\|\vec{x}\right\|}\left(\begin{array}{cc} x_{1} & -x_{2}\\ x_{2} & x_{1} \end{array}\right).\;$$

Note that the constraint on θ in (5) is imposed by assuming that the elbow joint can rotate within the limits [0, π]. Otherwise it can be relaxed.

**Extension due to collision with forearm**, ***E***_**2**_. If the object is reachable by the forearm, then we have:

(7) $$E_{2}(\vec{x})=\left\{F_{2}(\phi ,\vec{x}):\phi \in [-\phi_{\text{max}},0]\right\}\times J,$$

where

(8) $$F_{2}\left(\phi ,\vec{x}\right)=\frac{\vec{x}}{\lambda_{\vec{x}}(\phi )}-\rho \left[\frac{1}{\lambda_{\vec{x}}(\phi )}-1\right]M\left(\begin{array}{c} \text{cos }\phi \\ \text{sin }\phi \end{array}\right)$$

and $\lambda_{\vec{x}}\left(\phi \right)={\left(\rho^{2}+||\vec{x}{||}^{2}-2\rho ||\vec{x}||\text{cos }\phi \right)}^{\frac{1}{2}}$. The lower bound for ϕ in (7) is given by

(9) $$\phi_{\text{max}}(\vec{x})=\text{arccos}\left(\frac{\rho^{2}+\|\vec{x}{\|}^{2}-1}{2\rho \|\vec{x}\|}\right).$$

**Extension due to collision with foil**, ***E***_**3**_. Assuming that the foil is in contact with the object at a distance $d=||\vec{x}-{\vec{x}}_{h}||<h$ from the wrist, we get:

(10) $$E_{3}(\vec{x})=\left\{F_{3}(d,\alpha ,\vec{x}):d\in [d_{\text{min}},h],\alpha \in J\right\},$$

where

(11) $$\begin{array}{l} F_{3}\left(d,\alpha ,\vec{x}\right)={\vec{x}}_{e}+\frac{\text{sign}(\alpha )}{\beta_{\vec{x}}\|\vec{x}-{\vec{x}}_{e}\|}\left(I_{2}+dR_{\alpha }\right)(\vec{x}-{\vec{x}}_{e}),\;\\ \beta_{\vec{x}}={\left(1+d^{2}+2d\text{cos }\alpha \right)}^{\frac{1}{2}}, \end{array}$$

and *R*_α_ is the standard clockwise rotational matrix. In Equation (11)

(12) $${\vec{x}}_{e}=\frac{1}{2\|\vec{x}\|}M\left(\begin{array}{c} \|\vec{x}{\|}^{2}+\rho^{2}-\lambda_{\vec{x}}^{2}\\ -{\left[{\left(2\|\vec{x}\|\rho \right)}^{2}-{\left(\|\vec{x}{\|}^{2}+\rho^{2}-\lambda_{\vec{x}}^{2}\right)}^{2}\right]}^{\frac{1}{2}} \end{array}\right).$$

The lower bound for *d* in (10) is given by $d_{\text{min}}=\text{max}\left\{0,{\left[{\left(||\vec{x}||-\rho \right)}^{2}-{\text{sin}}^{2}\alpha \right]}^{\frac{1}{2}}-\text{cos }\alpha \right\}$.

### 2.3. Neural Network Generating Generalized Cognitive Maps in Hand-Space

To generate a GCM, we simultaneously (i) predict the objects' movements and (ii) simulate all possible subject's actions matched with the objects' movements. Both calculations must be done by the subject faster than the time scale of the dynamic situation (for more detail see Calvo et al., 2016). To account for this internal processing, besides the “real” time *t* in the workspace W, we introduce the “mental” time τ used for calculations in the hand-space H. For convenience, we also introduce the discrete time *n* ∈ ℕ_0_ related to the continuous time by τ = δ*n*, where δ is the time step.

There are several ways to solve problem (i) (see, e.g., Hong and Slotine, 1997; Riley and Atkeson, 2002; Villacorta-Atienza et al., 2010; Villacorta-Atienza and Makarov, 2013). For simplicity, we assume that the trajectories of all objects (except the subject, L) are given. Using these trajectories, we can evaluate images of the objects (e.g., the opponent's foil) in the hand-space (section 2.2.2). Then, we simulate all possible movements of the subject by means of a wave process initiated at the initial configuration $\left({\vec{x}}_{h}\left(0\right),\alpha \left(0\right)\right)\in \;H$.

In earlier works, we considered 2D internal representations of workspaces and postulated a constant velocity *c* for the wave of excitation spreading in the hand-space (Villacorta-Atienza et al., 2010, 2017; Calvo et al., 2017). The propagating wavefront simulates all possible movements of the hand (see below). In the 3D hand-space, the wrist joint can rotate with an angular velocity ω = *dα*/*dt* ∈ ℝ independent of the hand velocity $||\vec{v}||$ in the (*x*_1_, *x*_2_)-plane. Then, we impose the following constraint on the compound subject velocity in H:

(13) $$c^{2}=(1-\gamma_{0})\|\vec{v}(\tau ){\|}^{2}+\gamma_{0}\omega^{2}(\tau ),$$

where γ_0_ ∈ [0, 1] is the velocity bias. The value γ_0_ = 0 corresponds to a rigid wrist joint. The other limit γ_0_ = 1 describes the situation where the wrist flexion is the only available movement, i.e., the subject's upper limb is fixed. We note that formulation (13) is equivalent to fixing the kinetic energy of L.

To describe the wave dynamics, we design a neural network on the cylindrical lattice:

(14) $$\Lambda =\left\{\lambda =(i,j,k)\in {\mathbb{Z}}^{3}:\;i^{2}+j^{2}\leq r^{2},\;1\leq k\leq K\right\},$$

where *r* ∈ ℕ defines the spatial resolution in the plane (*x*_1_, *x*_2_), and *K* ∈ ℕ defines the resolution for the wrist flexion angle α. Thus, Λ is the *discrete version of the hand-space* H. On the lattice Λ, we define the neural network:

(15) $$\begin{array}{l} \frac{du_{\lambda }}{d\tau }=q_{\lambda }(f(u_{\lambda })+d_{0}((1-\gamma )\Delta_{x}+\gamma \Delta_{\alpha })u_{\lambda }),\;\lambda \in \Lambda ,\\ q_{\lambda }(\tau )=\{\begin{array}{l} 0\text{ if }\lambda \in \Omega (n)\cup L,\text{ with }(n-1)h<\tau \leq nh\\ 1\text{ otherwise}, \end{array}\\ \Omega (n)\leftarrow \Omega (n-1)\cup \{\lambda \in \Gamma (n):\;u_{\lambda }(nh)\in [0.4,0.7]\},\;\\ n=1,2,\ldots , \end{array}$$

where *u*_λ_ is the state variable describing the neuronal dynamics, *f*(*u*) = *u*(*u* − 0.1)(1 − *u*) is the nonlinear function providing the excitable dynamics of individual neurons, Δ_*x*_, Δ_α_ are the discrete Laplacians in the corresponding variables, Γ(*n*) is the set of neurons occupied by the extended objects at time instant *n*, and *L* is a small spheroid centered at λ_0_ (the discrete version of $\left({\vec{x}}_{h}\left(0\right),\alpha \left(0\right)\right)\in H$).

The dynamics of the neural network (15) admits propagation of spherical waves starting from the spheroid *L* (Calvo et al., 2018b). The initial spheroid sets the eccentricity of the wavefront defined by the bias parameter $\gamma =\gamma_{0}\frac{{\left(\rho +1\right)}^{2}K^{2}}{r^{2}|J{|}^{2}}$. Then, the wave propagates outwards, excites cells not occupied by obstacles, and creates effective objects when colliding with extended objects in the hand-space. The dynamically growing set Ω(*n*) describes effective objects at step *n*.

The dynamical system (15) is considered with Neumann boundary conditions on the cylinder border and extended objects. At τ = 0 (and hence *n* = 0), the neurons are set to *u*_λ_(0) = 0, ∀λ ∈ Λ \ *L*, *u*_λ_(0) = 1 ∀λ ∈ *L*, and Ω(0) = ∅. The diffusion coefficient *d*_0_ is adjusted to account for the compound subject velocity *c* (Calvo et al., 2018b).

### 2.4. Robot and Avatar Design

**Figure 2:**
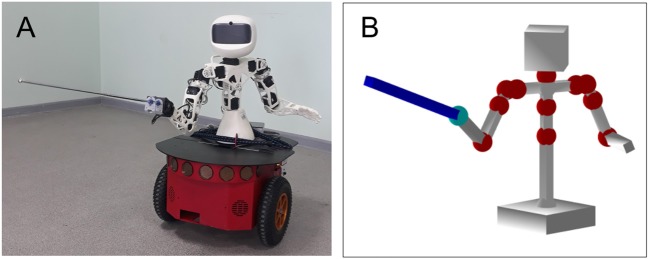
Experimental design. **(A)** Robotic fencer. A humanoid robot Torso (in white) is attached to a wheeled platform Pioneer 3DX (in red). **(B)** Avatar in virtual reality.

For testing and validating the theoretical results, we built a humanoid robot consisting of a robot Poppy Torso (upper part) attached to a wheeled platform Pioneer 3DX (Figure 2A). The wheeled platform provides the robot with the possibility to freely move in space, while the Torso enables manipulation of a foil.

To build the upper part of the robot, we used the open-source project Poppy Torso (Lapeyre et al., 2014; Duminy et al., 2016). The robot is based on Dynamixel smart servomotors and 3D printed plastic elements. The geometric dimension of the upper arms 15.5 cm (from the shoulder to the elbow), the forearms are 15 cm long (from the elbow to the foil hilt), and a toy foil is of 45 cm. To move the robot's body, we used a wheeled platform Pioneer 3DX (Adept Mobilerobotics, linear sizes l × w × h: 45.5 × 38.1 × 23.7 cm). An onboard computer (NUC, Intel) drives the robot through appropriate interfaces (USB and USB-COM).

To control the robot, we developed software called Avatar, which runs in a standalone PC. The Avatar serves as a bidirectional interface between the robot's body and its artificial “brain.” Thus, it provides the embodiment of the cognitive skills developed in simulations. The Avatar can work in two modes: (1) Driving the robot, and (2) Emulating the robot in virtual reality (Figure 2B).

In the first mode, the Avatar controls the servomotors of the robot. Thus, we can change the configuration of the robot's upper limbs and move it in space. At the initialization, the Avatar takes settings (main parameters such as e.g., the length of segments) from a text file, which allows flexible changes without reprogramming. The Avatar can also read in real-time the telemetric information of all servomotors. The software uses a client-server architecture based on TCP/IP for interacting with user programs simulating cognitive behaviors (artificial brain). The application programming interface allows controlling the robot movements at low and high levels. The low level allows selecting a motor and performing the desired rotation. At the high level, the user can send a trajectory for moving, e.g., the robot's arm ${\vec{x}}_{h}(t)={\left(x_{h1}(t),x_{h2}(t)\right)}^{T}$. Then, the Avatar solves the inverse kinematics problem.

(16) $$\begin{array}{l} \varphi =\text{arctan}2(x_{h2},x_{h1})-\phi_{\text{max}}({\vec{x}}_{h}),\;\\ \theta =\pi -\text{arccos}\left(\frac{\|{\vec{x}}_{h}{\|}^{2}-\rho^{2}-1}{2\rho }\right),\;\theta \in [0,\pi ], \end{array}$$

and applies the corresponding rotations to the servomotors in an automatic mode.

In the emulation mode, the Avatar builds a 3D model of the robot in a virtual environment (Figure 2B). Then, all the requested movements can be implemented by the virtual robot in the same way as would be done with the real robot. The Choregraphe program by the Aldebaran implements a similar functionality for the NAO robot (Pot et al., 2009; Shamsuddin et al., 2011). However, NAO is not suited for tasks considered in this work, in part, due to a significant deviation from anthropomorphic measures.

## 3. Results

### 3.1. Emergence of Generalized Cognitive Maps and Single Motor Actions

**Figure 3:**
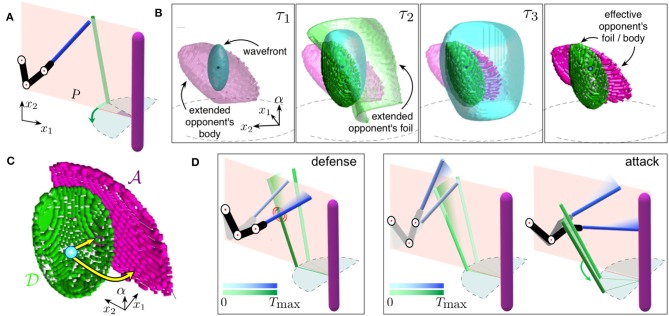
Emergence of cognitive map driving subject's actions. **(A)** Sketch of a combat situation. The subject's upper limb (in black) and the foil (in blue) are shown. The pink vertical bar represents the opponent with a foil (in green). **(B)** Generation of the GCM in the discretized hand-space Λ. Three successive snapshots (τ_1_, τ_2_, and τ_3_) and the final GCM (right subplot) are shown. **(C)** Two typical trajectories (yellow curves) of the subject movement in the discrete hand-space. Arrows starting at blue point and ending at the pink/green area correspond to an attack/defense movement (sets A and D). **(D)** Implementation of the trajectories shown in **(C)** in the workspace. Left: Execution of the defensive trajectory. Right: Execution of the attack trajectory (the color darkness corresponds to the time course).

Let us first consider how a GCM can be constructed for a novel dynamic situation. Figure 3A illustrates a simple combat scene similar to that shown in Figure 1A. The upper limb and the foil of the cognitive fencer (on the left) are aligned within the main plane. Its opponent (on the right) moves its foil with a constant angular velocity by a circular displacement of the hand, from right to left. The goal of the subject is to either defend (i.e., to stop the opponent's foil) or attack (i.e., to hit the opponent's body by the foil tip). Such decision-making goes through the construction of a generalized cognitive map.

Figure 3B shows three successive snapshots at time instants τ_1_ < τ_2_ < τ_3_ illustrating the process of building the GCM in the discrete hand-space Λ (see section 2.3). The traveling wavefront (light-blue) explores the environment containing the extended opponent's body (snapshot τ_1_). Note that the opponent's body does not move and the corresponding extended object has a fixed shape in all snapshots. In contrast, the opponent's foil crosses plane *P* in a certain time interval and hence its representation in Λ changes in time (it is present in snapshot τ_2_ only).

Let λ_0_ ∈ Λ be the point representing the limb configuration at τ = 0, i.e., the discrete version of C(L)∈H (see Equation 3). We then can express the process of generation of a GCM given by Equation (15) as the map:

(17) $$G_{\lambda_{0}}:\Lambda \to {\mathbb{N}}_{0},\text{ s.t. }G_{\lambda_{0}}(\lambda_{0})=0,\;G_{\lambda_{0}}(\lambda )>0\text{ for all }\lambda \neq \lambda_{0}.$$

*G*_λ_0__(λ) stores the time taken by the subject to modify the configuration of its limb from λ_0_ to λ, following the course of the wave propagation.

While propagating, the wavefront hits the extended opponent's body and foil, which produces static effective objects Ω(1) ⊆ ⋯ ⊆ Ω(*n*_max_) = Ω that can be reached by the subject's foil. The process (see Equation 15) creates the GCM when the wave propagation ends:

(18) $$M={\left\{\left(\lambda ,G_{\lambda_{0}}(\lambda )\right)\right\}}_{\lambda \in \Lambda }\subset {\mathbb{Z}}^{4}.$$

Note that for each λ ∈ Ω, *G*_λ_0__(λ) ∈ ℕ represents the time instant when the subject's foil either stops the opponent's foil or hits the opponent's body at location λ. We thus can divide this set into sets for a defense D and for an attack A (dark green and dark pink sets in Figure 3B, respectively):

(19) A∪D=Ω.

Figure 3C shows two representative examples of the subject movements in the hand-space. One of them (yellow arrow ending in the green area) corresponds to a defense action λ∈D, i.e., the subject stops the opponent's foil, whereas the other (yellow arrow ending in the pink area) describes an attack λ∈A, i.e., the foil tip hits the opponent's body.

We can now unfold the trajectories from the hand-space to the workspace (i.e., solve the inverse kinematic problem). Figure 3D shows two combat actions corresponding to a defense and an attack. In the first case (Figure 3D, left), the subject lowers his foil and stops the opponent's attack. In the second case (Figure 3D, right), the subject performs a “two-step” action. First, he lifts the foil and then moves it down, simultaneously rotating his wrist. Thus, the subject circumvents the opponent's foil and then hits the opponent's body.

### 3.2. Cognitive Substrate for Building Strategies in Dynamic Situations

**Figure 4:**
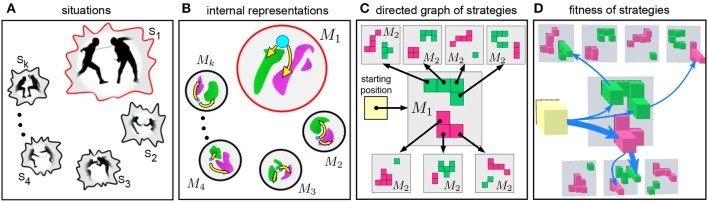
Building optimal strategies in dynamic situations. **(A)** A fencer learns to actuate in different dynamic situations while training. **(B)** Sketches of generalized cognitive maps of the learned situations. Blue dot represents the subject's upper limb and the foil. Yellow paths to pink and green regions correspond to attack and defense actions, respectively. **(C)** Sketch of two-step strategies. The subject invokes a first order map *M*_1_ from the starting position and gets several options for the first movement. Then, each movement leads to a second order map *M*_2_ with new possible movements. The strategy is built by selecting one of the pathways in the directed graph. **(D)** Each action has a fitness (imminence, lethality, and versatility) denoted by the height of colored squares. This allows selecting the most suitable strategies (blue arrows).

To master different skills of defense and attack, a fencer learns to actuate in different situations *S*_1_, …, *S*_*m*_ simulating parts of real combat (Figure 4A). Such learning shapes generalized cognitive maps *M*_1_, …, *M*_*n*_ describing the internal representation of each situation (Figure 3). Thus, the time dimension of the perceived situations is compacted into the fourth dimension of *M* while the combat-relevant spatiotemporal events are mapped into a virtual *collision* space (Figure 4B).

#### 3.2.1. From Single Movements to Series of Actions

As we discussed above, the collision space is configurational. The whole subject's limb and the foil are represented by a single point in H and hence in Λ (blue dot in Figures 3C, 4B), while the opponent's body and the foil are augmented and mapped by the wave-process into static effective objects (green and pink areas in Figures 3C, 4B). Such virtual objects represent collisions with the subject's foil. Thus, depending on the fighting skills, the fencer gets potential options to attack or to defend by following a trajectory from the blue dot to either red (A) or green area (D) (yellow arrows in Figures 3C, 4B).

From the mathematical viewpoint, a GCM, *M*_1_(λ_0_), can be defined by the mapping *G*_λ_0__ (see Equation 18). Now, once the agent is able to move from λ_0_ to λ_1_ ∈ Ω_1_ in time *G*_λ_0__(λ_1_), a second GCM can be generated by using λ_1_ as the initial limb configuration: *M*_2_(λ_0_, λ_1_) = {(λ,*G*_λ_1__(λ))}_λ∈Λ_. Such a process can be continued, and we get the chain

(20) $$\begin{array}{l} \;\lambda_{0}\mapsto M_{1}∋(\lambda_{1},n_{1}),\;\lambda_{1}\mapsto M_{2}∋(\lambda_{2},n_{2})\;,\\ \lambda_{2}\mapsto M_{3}∋(\lambda_{3},n_{3}),\;\ldots ,\;\lambda_{k-1}\mapsto M_{k}∋(\lambda_{k},n_{k}), \end{array}$$

where *n*_*k*_ = *G*_λ_*k*−1__(λ_*k*_) is the time taken to move the subject's limb from λ_*k*−1_ to λ_*k*_, and *M*_*k*_ = {(λ,*G*_λ_*k*−1__(λ))}_λ∈Λ_ is the *k*-th order GCM. Thus, the fencer can design a route consisting of a chain of (*k* + 1) points in the hand-space λ_0_ → λ_1_ → ⋯ → λ_*k*_. Such a route allows reproducing a series of concatenated actions according to a certain strategy. Since each GCM enables several different movements with the foil (e.g., two yellow curves in each GCM shown in Figure 4B), each movement, λ_*i*−1_ → λ_*i*_, can be followed by a series of next movements. This leads to the emergence of a strategy graph and a reach variety of combat repertoires.

#### 3.2.2. Semantic Description of Strategies Over Cognitive Substrate

Let us now consider the process of building strategies in detail. Figure 4C shows the sketch of planning a two-step action. When the opponent initiates the first movement from a specific starting position, the subject predicts the evolution of the situation (yellow square) and invokes the first GCM (Figure 4C, gray square *M*_1_). Green and pink Tetris-like pieces in map *M*_1_ illustrate the sets $D_{1}$ and $A_{1}$, respectively. We remind that moving to one of the pieces in $D_{1}$ or $A_{1}$ in the hand-space corresponds to a defense or attack action in the workspace.

Each specific square in the first order map (Figure 4C) *m*_1_: = (λ_1_, *n*_1_) ∈ *M*_1_ can be considered as a motor-motif, i.e., an essential motor actions of the subject (Colome and Torras, 2014; Makarov et al., 2016; Calvo et al., 2018a). We now can take one motor-motif *m*_1_ and use it as a new situation for generating the second motor-motif through a second order GCMs (Figure 4C, black arrows pointing to gray squares marked as *M*_2_). Each of these GCMs provides several options for the second motif (Figure 4C, Tetris-like pieces in maps *M*_2_). Such an iteration can be repeated and thus we get a sequence of motor-motifs:

(21) $$m_{1}\to m_{2}\to \cdots \to m_{k}.$$

Sequences of the form (21) define diverse semantic contents of the possible chained actions of the subject. For example, the simplest defense-attack chain is given by (λ_1_, *n*_1_) → (λ_2_, *n*_2_), where $\lambda_{1}\in D_{1}$ and $\lambda_{2}\in A_{2}$. Note that there are many such chains even for a single given situation (Figure 4C). Now the subject can learn different semantic chains and perform series of motor-motifs.

#### 3.2.3. Strategy Fitness

Each piece in a Tetris-like map (Figure 4C) represents an action with some particular features such as imminence, lethality, and versatility. We then can assign the corresponding fitness values to all pieces in the maps (Figure 4D). Now taking a particular chain of motor-motifs *m*_1_ → … → *m*_*k*_, the subject can evaluate its compound fitness and thus select the most suitable strategy according to his motivation by maximizing, e.g., the safety (thick blue arrow in Figure 4D).

Let us now introduce definitions for a two-step strategy *d*_1_ = (λ_1_, *n*_1_) → *a*_2_ = (λ_2_, *n*_2_), with $\lambda_{1}\in D_{1}$ and $\lambda_{2}\in A_{2}\left(\lambda_{1}\right)$.

- *Imminence:* It is the relative time taken by a chain of motor-motifs:
  (22) $$\begin{array}{l} F_{\text{imm}}\left(\lambda_{1}\right):=1-\frac{{\text{min}}_{\lambda_{2}\in \Omega_{2}}\left\{G_{\lambda_{1}}\left(\lambda_{2}\right)\right\}}{{\text{max}}_{\lambda_{1}^{\prime }\in \Omega_{1}}\left\{\text{min}\left\{G_{\lambda_{1}^{\prime }}\left(\lambda_{2}\right)\right\}\right\}}. \end{array}$$
- *Lethality:* It is the mean injury to the opponent's body made by a chain of motor-motifs:
  (23) $$F_{\text{let}}(\lambda_{1}):=\frac{\sum_{\lambda_{2}\in A_{2}(\lambda_{1})}L(\lambda_{2})}{\left|A_{2}(\lambda_{1})\right|},$$
  where *L* : Λ → [0, 1] is the operator defining the opponent's resistance to injury. Here, we use a piecewise linear function increasing from 0 (null lethality) in the opponent's feet to 1 (highest lethality) in the opponent's neck, and decreasing again to 0.4 in the opponent's head.
- *Versatility:* It is the relative size (i.e., the relative cardinality) of the set of available attacks:
  (24) $$\begin{array}{l} F_{\text{ver}}\left(\lambda_{1}\right):=\frac{\left|A_{2}\left(\lambda_{1}\right)\right|}{{\text{max}}_{\lambda_{1}^{\prime }\in \Omega_{1}}\left\{\left|A_{2}\left(\lambda_{1}^{\prime }\right)\right|\right\}}. \end{array}$$

We note that all strategy features are defined over the point λ^1^ in the first order map *M*_1_. Finally, the compound fitness of a strategy is given by:

(25) $$F(\lambda_{1})=\alpha_{\text{imm}}F_{\text{imm}}(\lambda_{1})+\alpha_{\text{let}}F_{\text{let}}(\lambda_{1})+\alpha_{\text{ver}}F_{\text{ver}}(\lambda_{1}),$$

where the factors α define the bias between the strategy features. Such a bias depends on the subject's motivation. For example, if the combat comes to its end, the weights of imminence and lethality can be raised, whereas at the combat beginning one can maximize imminence and versatility.

### 3.3. Two-Step Parry-Riposte Strategies

Let us now illustrate how a complex combat strategy known as parry-riposte can be built by using the approach shown in Figure 4. In this case, the subject uses the strength of his foil to block the opponent's attack (parry), and then he begins a counter-attack (riposte) with the aim of winning the combat.

#### 3.3.1. Optimization Over Two-Symbol Semantic Chains

**Figure 5:**
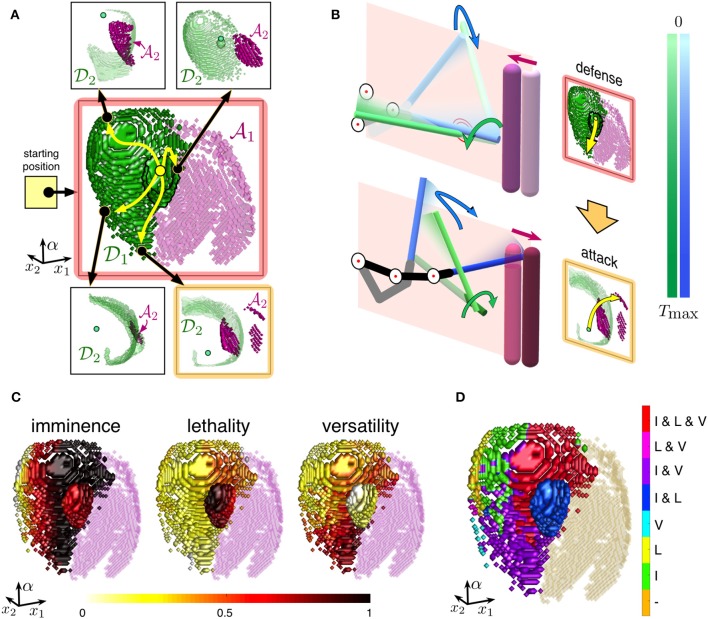
Semantic of parry-riposte strategies. **(A)** Strategies in the hand-space following Figure 4C (initial situation as in Figure 3). In the parry step, the subject uses a defensive movement *d*_1_ reaching some point λ_1_ in the set $D_{1}$ (green region). In the riposte step, the subject invokes one of the secondary maps and initiates an attack (pink points in $A_{2}$). **(B)** Implementation in the workspace of a strategy from the map highlighted in yellow in **(A)**. First, the subject's foil deflects the opponent's foil (parry). Then, the attack action is executed (riposte). **(C)** Fitness of semantic strategies presented over the set $D_{1}$. The color represents features of the defensive actions leading to: short/long attack trajectories (imminence), more/less dangerous attacks (lethality), and different/similar offensive actions (versatility). **(D)** Semantic meaning of different motor-motifs represented over the set $D_{1}$. Color represents different combinations of the attributes (I, imminence; L, lethality; V, versatility).

We consider the initial situation shown in Figure 3A. The opponent takes a step forward and makes an offensive circular movement by his foil. As a response, the subject invokes the cognitive map corresponding to this situation (Figure 3C). In this case, however, we are interested in the defense movements only and hence select one of the points from the set $D_{1}$ (Figure 5A, green surface in the first map highlighted in red). This defines the parry step *d*_1_ = (λ_1_, *n*_1_) ∈ *M*_1_, with $\lambda_{1}\in D_{1}$, similar to the defense movement shown in Figure 3D. However, now we consider different options for blocking the opponent's foil, i.e., trajectories ending at different green points of the map $d_{1}^{\prime }\in M_{1}$.

Each of the defense movements $d_{1}^{\prime }$ gives rise to a new situation after the parry step (see Equation 21) and a secondary cognitive map (Figure 5A, see also Figure 4C). Then, the subject can select an attack movement, i.e., draw a path in the hand-space Λ to a pink point $\lambda_{2}\in A_{2}$ in one of the maps *M*_2_. Such a path defines a riposte step *a*_2_ = (λ_2_, *n*_2_), which ends with hitting the opponent's body by the subject's foil. Figure 5B illustrates the sequence of the subject's and opponent's actions in the workspace W. First, the opponent takes a step forward and attacks. The subject deflects the opponent's attack, and the opponent takes a step back while the subject makes an offensive action and hits the opponent.

As above-discussed, the subject has a variety of two-symbol chains $\left\{d_{1}\to a_{2}\;:\lambda_{1}\in D_{1},\lambda_{2}\in A_{2}\left(\lambda_{1}\right)\right\}$. We now can evaluate the fitness of each semantic chain for a given defensive movement *d*_1_ = (λ_1_, *n*_1_). Figure 5C shows the imminence, lethality, and versatility of different strategies. It is worth noting that the strategy features achieve their maxima at different parts of the set $D_{1}$. Thus, as it frequently occurs in real combat, there is no global optimum, and the fencer should resort to a complex optimization, depending on his motivation. For illustration purpose, in Figure 5B, we have chosen the strategy maximizing the imminence and versatility with low lethality (Figure 5A, highlighted in yellow).

#### 3.3.2. Linking Motor-Motifs to Semantic Meaning

The measures of the strategy fitness now can be used to define the semantic description of the strategies. We continue working with two-step actions as above and use the definition of motor-motifs. Then, each motor-motif is a single strategy, which generates certain fitness measures (Figure 5C).

Now, each point in the defensive set $D_{1}$ defines some motor-motif. If a particular fitness measure exceeds a threshold (set to 0.3 in simulations), then we link the motor-motif to the corresponding attribute. In our case, each motor-motif can have up to three attributes: I for imminence, L for lethality, and V for versatility. Figure 5D shows combinations of the attributes for different motor-motifs. Thus, the fencer can now get access to the semantic meaning of each action. Due to significant dimension reduction, such a meaning can be easily stored and retrieved on purpose.

### 3.4. Validation of Approach in Humanoid Robot

Let us illustrate the above-described theoretical approach in the developed humanoid robot (see section 2). We use again the parry-riposte combat situation shown in Figure 5. However, now we select another, more aggressive riposte strategy than shown in Figure 5B. This is achieved by checking the semantic meaning shown in Figure 5D and then by choosing the corresponding motor-motifs in Figure 5A.

**Figure 6:**
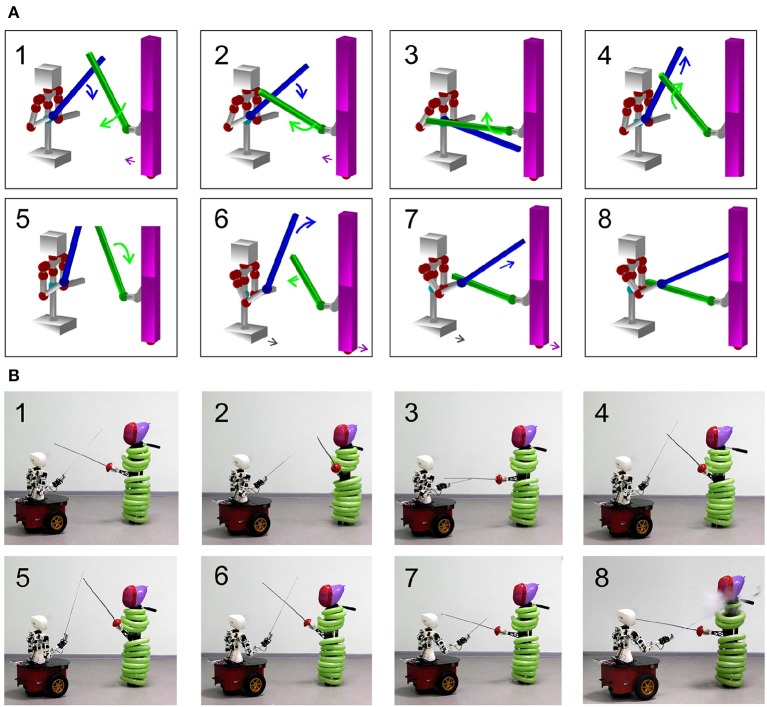
Experimental validation of a parry-riposte strategy. **(A)** Implementation of the strategy in virtual environment. **(B)** Same as in **(A)** but executed by the robot.

As has been mentioned, the Avatar software allows emulating a combat situation in virtual reality. Figure 6A shows a series of snapshots of the Avatar. The Avatar first deflects the opponent's foil, and then conducts an effective attack and hits the opponent.

Finally, we implemented the same dynamics in the humanoid robot. Figure 6B illustrates the sequence of snapshots. The robot first makes a defense movement and then attacks the opponent. To get a simple marker of hitting the opponents by the subject's foil, we used balloons. In the last snapshots, one can see how one balloon explodes, which confirms a point scored by the robot in this combat situation.

## 4. Conclusions

The cognitive-motor skills exhibited by humans in fast dynamic situations are far beyond the abilities of modern humanoid robots. Object manipulation is one of the prominent examples widely observed in different sports. In this work, we have considered the combat game of fencing, which, besides fast manipulation, includes precise strategic planning. We have provided and experimentally validated a novel approach to building strategies on an abstract cognitive level. The procedure uses the theory of generalized cognitive maps generated in a configuration space, the so-called hand-space. We have shown how GCMs can be constructed in a discrete 3D lattice representing three degrees of freedom of an upper limb handling a foil. A neural network simulates the process of the parallel exploration of different movements of the fencer. It thus transforms the dynamic combat situation into a static 4D map encapsulating all relevant events.

The resulting 4D cognitive map can be readily used for planning actions. However, what is more important, it enables a possibility to construct multi-action strategies in an abstract semantic way. Different GCMs can be chained, aiming at human-like multilevel decision-making. Starting from an initial position of combat, the fencer can select one of the motor-motifs (a point in the corresponding GCM) for the next movement. In turn, this leads to a new situation, which is also described by a GCM. Such a secondary GCM provides a variety of subsequent actions. This way, the fencer generates a chain of symbols, e.g., (*d*_1_, *a*_2_, *a*_3_, *d*_4_, …) describing the sequence of defense and attack movements.

We then have introduced the fitness depicting each strategy (symbolic chain) in terms of the imminence (velocity of actions), lethality (effect over the opponent), and versatility (variety of available movements). Note that the fencer can use the strategy fitness to optimize his actions depending on the motivation (a higher level of cognition). For example, at the beginning of combat, the fencer can use imminence and versatility as main attributes, whereas, at the end, the lethality may be the goal. We then confirmed our theoretical modeling by using the robot Torso, which has 3DoF in its upper limbs. To gain versatility, we have developed an avatar of the robot, which enables close to real simulations of different fencing situations. The experimental results validated the approach.

Concluding, the GCM theory and its generalization to the semantic abstraction provide a functional bridge between straightforward cognition, dealing with direct interaction in the workspace, and abstract cognition, whose impact over the subject's behavior is less immediate but much more profound. The semantic level of strategy description presented here takes a step forward to the latter ambitious goal.

## Data Availability Statement

The datasets generated for this study are available on request to the corresponding author.

## Author Contributions

CC, JV-A, and VM contributed conception, design of the study, and the mathematical analysis. CC, JV-A, and SD-H performed numerical simulations. MK designed and developed the Avatar software. SL and IP developed the robot and performed experiments. AS-J performed the statistical analysis. VM wrote the manuscript. All authors contributed to the manuscript revision, read, and approved the submitted version.

### Conflict of Interest

The authors declare that the research was conducted in the absence of any commercial or financial relationships that could be construed as a potential conflict of interest.
